# Interaction between dual specificity phosphatase-1 and cullin-1 attenuates alcohol-related liver disease by restoring p62-mediated mitophagy

**DOI:** 10.7150/ijbs.81447

**Published:** 2023-03-21

**Authors:** Ruibing Li, Zhe Dai, Xiaoman Liu, Chunling Wang, Jia Huang, Ting Xin, Ying Tong, Yijin Wang

**Affiliations:** 1 School of Medicine, Southern University of Science and Technology, Shenzhen, Guangdong, China.; 2 Department of Clinical Laboratory Medicine, The First Medical Centre, Medical School of Chinese People's Liberation Army, Beijing, China.; 3 Department of Cardiology, Tianjin First Central Hospital, Tianjin 300192, China.

**Keywords:** Alcohol-related liver disease, DUSP1, CUL1, mitophagy

## Abstract

Besides abstinence, no effective treatment exists for alcohol-related liver disease (ALD), a dreaded consequence of alcohol abuse. In this study, we assessed the roles on ALD of dual specificity phosphatase-1 (DUSP1), an hepatoprotective enzyme, and Cullin-1 (CUL1), a member of the E3 ubiquitin ligase complex that exerts also transcriptional suppression of mitochondrial genes. Alcohol treatment downregulated hepatic DUSP1 expression in wild-type mice. Notably, DUSP1 transgenic (*Dusp1^Tg^*) mice showed resistance to alcohol-mediated hepatic dysfunction, as evidenced by decreased AST/ALT activity, improved alcohol metabolism, and suppressed liver fibrosis, inflammation, and oxidative stress. Functional experiments demonstrated that DUSP1 overexpression prevents alcohol-mediated mitochondrial damage in hepatocytes through restoring mitophagy. Accordingly, pharmacological blockade of mitophagy abolished the hepatoprotective actions of DUSP1. Molecular assays showed that DUSP1 binds cytosolic CUL1 and prevents its translocation to the nucleus. Importantly, CUL1 silencing restored the transcription of p62 and Parkin, resulting in mitophagy activation, and sustained mitochondrial integrity and hepatocyte function upon alcohol stress. These results indicate that alcohol-mediated DUSP1 downregulation interrupts DUSP1/CUL1 interaction, leading to CUL1 nuclear translocation and mitophagy inhibition via transcriptional repression of p62 and Parkin. Thus, targeting the DUSP1/CUL1/p62 axis will be a key approach to restore hepatic mitophagy as well as alleviate symptoms of ALD.

## Introduction

Alcohol-related liver disease (ALD) is characterized by pathological changes in hepatic tissue resulting from excessive alcohol use. Hallmarks of ALD include hepatic steatosis (fatty liver) due to build-up of fat deposits inside liver cells, alcoholic hepatitis, resulting from acute inflammation, with progressive fibrosis and development of alcoholic cirrhosis manifesting at advanced stages. More than 30% of patients with ALD have been diagnosed with hepatitis C, and ~50% have gallstones. Importantly, patients with alcoholic cirrhosis may develop severe complications such as liver cancer, intestinal bleeding, and kidney failure. The only effective treatment for ALD is alcohol abstinence. However, this seems to have no significant prognostic benefits for patients with alcohol-associated cirrhosis. Thus, investigation of the molecular basis behind ALD may offer novel approach for the prevention and treatment of alcohol-related hepatic injury.

Dual specificity phosphatase-1 (DUSP1), as a phosphatase, plays an important role in mediating dephosphorylation and inactivation of MAPK family members such as ERK, JNK, and p38 1. By mitigating the contribution of JNK and p38 signaling to the inflammatory response, DUSP1 was shown to act as an anti-inflammatory factor in several conditions and diseases, including colonic inflammation 2, airway inflammation 3, polymicrobial peritonitis 4, septic cardiomyopathy 5, neuroinflammation 6, and carbon tetrachloride-induced liver injury 7. Besides, recent studies also reported the beneficial effects of DUSP1 on lipopolysaccharide-induced hepatic oxidative stress 8, stress-related liver injury 9, and toxin-induced hepatic dysfunction 10. However, the whether DUSP1 confers also hepatoprotection against ALD remains undetermined.

Mitochondrial damage, and especially impaired oxidative phosphorylation, are considered main contributing factors in the pathogenesis and progression of ALD 11, 12. Accordingly, mitochondria-targeted therapies have been found to improve liver function in rodent models of ALD 13-15. Under stress conditions, mitochondrial damage is counteracted by activation of an endogenous repair mechanism, which coordinates mitochondrial division/fusion and biogenesis 16-20. Another crucial process controlled by the MQC is mitochondrial autophagy (mitophagy), a PINK1/Parkin pathway-dependent process that serves to recycle the components of defective mitochondria to stabilize the mitochondrial network 21-24. Studies from our laboratory 25 and others 26, 27 have confirmed the protective effects of mitophagy in the setting of ALD. However, in ALD mitophagic activity is significantly inhibited due to as yet undefined mechanisms 28. Recent studies have elucidated the correlation between decreased DUSP1 expression and defective mitophagy in conditions such as cardiac ischemia/reperfusion injury 29 and diabetic nephropathy 30. This evidence led us to speculate that DUSP1 downregulation contributes to the development of ALD through impaired mitophagy.

Cullin-1 (CUL1), a member of the SKP1-Cullin 1-F-box protein (SCF) E3 ubiquitin-protein ligase complex, has been identified as a ubiquitination substrate of Parkin 31, 32. A well-characterized role of ubiquitinated CUL1 is to promote the activation of Cyclin-E, a mechanism involved in the proliferation of colorectal 33, breast 34, and prostate 35 cancer. In addition to this pro-tumorigenic role, recent research uncovered an inhibitory effect played by CUL1 on the expression of genes involving the structure and function of mitochondria 36. Indeed, abnormal CUL1 expression has been reported to be a potential factor inducing mitochondrial stress 37. Along these lines, impaired mitochondrial accumulation has been also observed in response to CUL1 overexpression 38. From this evidence, we also asked whether CUL1 regulates hepatic transcription of mitophagy-related genes in ALD. Thus, based on bioinformatics analysis predicting the interaction between DUSP1 and CUL1, the task of our present research was to figure out whether DUSP1 protects the liver against alcohol-related injury through preventing CUL1-mediated mitophagy suppression.

## Materials and Methods

### Animal experiments

DUSP1 transgenic mice (*Dusp1^Tg^*) and their wild-type (WT) littermates were bred on an FVB/N background (FVB.129P2-Abcb4tm1Bor/J) kindly offered by Prof. Hao Zhou (University of Wyoming) 29. Alcohol-related liver injury was induced as previously described 39 through feeding an ethanol diet (5% ethanol; F1258SP; Bio-Serv) for 8 weeks. The control group was treated with a liquid control diet also for 8 weeks.

### Histology and immunohistochemistry

Samples of liver tissue were treated with 4% paraformaldehyde. Then, 5 μm thick paraffin sections were generated, which was treated with hematoxylin and eosin (HE) to observe the hepatic morphology. Besides, immunohistochemistry (IHC) was performed on 5 μm thick paraffin sections which were stained with TGFβ (1:500; #ab189778, Abcam) according to a standard protocol 40.

### Immunoblotting

For immunoblot analysis of protein expression, liver tissues and hepatocytes were lysed in SDS-lysis buffer and transferred to 0.45 μm nitrocellulose membranes (10600002, Amersham) 41. Immunoblots were blocked by 5% non-fat milk followed by incubation with primary antibodies, including DUSP1 (1:1000, #sc-373841, Santa Cruz Biotechnology, Inc.), Cullin-1 (1:1000, #ab75817, Abcam), PINK1 (1:1000, #ab23707, Abcam), Parkin (1:1000, #ab77924, Abcam), and LC3II (1:1000, #ab192890, Abcam). Chemiluminescent signals were developed with Clarity Western ECL substrates (170-5061, Bio-Rad), and images were visualized by Amersham Imager 600 (GE Healthcare). The integrated optical intensity of each band was measured by Image-Pro Plus 6 software (Media Cybernetics) 42.

### Co-immunoprecipitation

Co-immunoprecipitation (Co-IP) experiments were adapted from our previous studies 43. For analyzing the interaction between DUSP1 and CUL1, cells were lysed in IP buffer, needled 20 times, and centrifuged at 12,000 g for 15 min 44. The samples were then incubated with 50 μl EZview Red Anti-Myc Affinity Gel slurry (E6654, Sigma-Aldrich) for 16 hr at 4 °C. After centrifugation, supernatant was mixed with membrane protein solubilization buffer. Then, SDS-PAGE was performed and immunoblotting was conducted to observe the bands.

### Immunofluorescence

Liver tissues and hepatocytes were treated with 4% PFA/PBS for 30 min at room temperature. Then, samples were treated with 0.25% Triton-X100/PBS for 30 min, followed by the incubation with 4% BSA/PBS for 45 min 22. Primary antibodies against MMP9 (1:1000, #ab58803, Abcam) and Cullin-1 (1:1000, #ab202555, Abcam) and suitable fluorescently-labeled secondary antibodies were sequentially incubated for 2 h, and nuclear staining performed with 5 μg/ml Hoechst 33342 before mounting in Mowiol 4-88 reagent (81381, Sigma). Fluorescence images was captured with a Zeiss 880 confocal microscope using a 100X oil objective, and acquired with same laser output and gain 45.

### Real-time quantitative PCR

Cells or liver tissues (~40 mg) were homogenized in TRIzol reagent (T9424, Sigma) and total RNA was isolated 46. RNA from each sample were used to synthesize cDNA. Quantitative gene expression data were acquired on a Bio-Rad CFX96 real-time PCR system. The primers for each analyzed gene were: Beclin1, F: 5′-TTTTCTGGACTGTGTGCAGC-3′, R: 5′-GCTTTTGTCCACTGCTCCTC-3′; p62, F: 5′-AAGTCAGCAAACCTGACG-3′, R: 5′-CCATCTGTTCCTCTGGCT-3′; PINK1, F: 5′-GAGCAGACTCCCAGTTCTCG-3′, R: 5′-GTCCCACTCCACAAGGATGT-3′; IL6, F: 5′-TAGTCCTTCCTACCCCAATTTCC-3′, R: 5′-TTGGTCCTTAGCCACTCCTTC-3′; β-actin, F: 5′-AGTGTGACGTTGACATCCGT-3′, R: 5′-GCAGCTCAGTAACAGTCCGC-3′; Parkin, F: 5′-AGTGTGACGTTGACATCCGT-3′, R: 5′-ACACGGCAGGGAGTAGCCAAGTTG-3′; CUL1, F: 5′-TTGCAAAGGGCCCTACGTT-3′, R: 5′-CGTTGTTCCTCAAGCAGACG-3′; NLRP3, F: 5′-AAGGGCCATGGACTATTTCC-3′, R: 5′-GACTCCACCCGATGACAGTT-3′; IL1β, F: 5′-CACGATGCACCTGTACGATCA-3′, R: 5′-GTTGCTCCATATCCTGTCCCT-3′; DUSP1, F: 5′-GGATATGAAGCGTTTTCGGCT-3′, R: 5′-GGATTCTGCACTGTCAGGCA-3′.

### Primary hepatocyte isolation and treatment

Male mice (10-week-old) were anaesthetized with 1% sodium pentobarbital (10 μl/g body weight), and the liver was perfused with perfusion buffer while digested with collagenase (C6885, Sigma) in buffer until the tissue became soft 47. The resulting preparation was filtered with a 70 μm cell strainer to remove debris. Cells were treated with ice-cold PBS, centrifuged at 150 g for 5 min, and seeded in 60-mm dishes (1 x 10^6^ cells/dish) pre-coated with collagen (C7661, Sigma). Hepatocytes were then grown until 80% confluence and then incubated with 100 mM ethanol for 24 h 39. Control cells were left untreated/treated with PBS.

### Serum and liver biochemistry and ELISA

Serum was collected from blood by centrifugation at 1,200 g for 15 min at 4 °C. Livers were homogenized by Precellys 24 ceramic beads, and the organic phase was dried under N^2^ and dissolved in ethanol 48. TC and TG levels in serum and liver were respectively measured using Cholesterol Assay and Triglyceride Assay kits (Shanghai Kehua Bio-engineering, China). Serum ALT (LE-M0477, Lai Er Bio-tech), serum AST (LE-M0568, Lai Er Bio-tech), serum or liver SOD (#ab285309, Abcam), serum or liver GSH (#MOEB2568, AssayGenie), liver MDA (#MOEB2496, AssayGenie), liver 4-NHE (#A76021, Antibodies.com), liver CAT (#CSB-E14190, CUSABIO), liver ADH (#A4731, Antibodies.com), liver ALDH (#MBS726168, MyBioSource, Inc.), and liver CYP2E1 (#CSB-EL006425MO, CUSABIO) contents were quantified by the indicated ELISA kits according to the manufacturers' instructions 49. Commercial kits were also used to detect glucose (Mouse Glucose Assay Kit, #81692, Crystal Chem) and lactic acid (Mouse Dextro-lactic acid ELISA Kit, #E03D0242, American Research Products Inc.) in hepatocyte culture media according to the manufacturers' instructions.

### siRNA transfection

Hepatocytes were cultured in DMEM Growth Medium-2 (Lonza) in 24-well plates until 60-80% confluence. Mouse CUL1 siRNA (#AM16708) and negative control siRNA (both purchased from ThermoFisher Sci.) were transfected using Lipofectamine RNAiMax reagent (Invitrogen). Ninety-six hours after transfection, hepatocytes were used for functional experiments.

### Mitochondrial membrane potential and ROS detection

Fluorescence-based measurements of mitochondrial membrane potential and intracellular ROS generation were performed as previously described 50. In brief, following treatment, hepatocytes were incubated with the potentiometric JC-1 sensor (Mitochondrial Membrane Potential Detection Kit, #30001, Biotium). For ROS measurements, fresh liver sections or primary hepatocytes were incubated with the cellular ROS indicator DHE (#PD-MY 003, MedChemExpress), cytoplasmic ROS indicator H2DCFDA (#HY-D0940, MedChemExpress), and mitochondrial ROS indicator MitoSOX RED (HY-D1055, MedChemExpress) based on methods provided by manufacturers 51. Fluorescence images were acquired with same laser output and gain for control and treated samples on a Zeiss 880 confocal microscope using a 100X oil objective 52.

### Electron microscopy

Hepatocytes and liver tissues were treated with 5% glutaraldehyde and 2% paraformaldehyde in 0.1 M sodium cacodylate buffer, pH 7.2. Samples were then teased apart and placed in 2.5% glutaraldehyde in 0.1 M sodium cacodylate buffer and then were stored in a 4 °C fridge overnight 53. Samples were processed and imaged at the Vanderbilt Univ. Cell Imaging Shared Resource (CISR) core facility 42. Images were analyzed in blinded fashion by an experienced electron microscopist. The magnification of the images was similar in all 3 groups.

### Mitophagy detection and ATP measurement

The mito-Keima reporter, a mitochondria-specific, pH-sensitive fluorescent probe, was transfected into primary hepatocytes to evaluate mitophagy 45. To observe the changes of mito-Keima in hepatocytes, we calculated the area of dots with a high 561/457 nm fluorescence ratio. Intracellular ATP concentration was analyzed using an ATP Bioluminescence Assay Kit HS II (#11699709001, Merck KGaA).

### Statistical analysis

Two-tailed Student's t-tests were performed in GraphPad software to compare means between two groups. One-way ANOVA was performed when two or more groups were compared. P < 0.05 was considered significant.

## Results

### DUSP1 overexpression attenuates alcohol-mediated hepatic dysfunction

**Figure 1:**
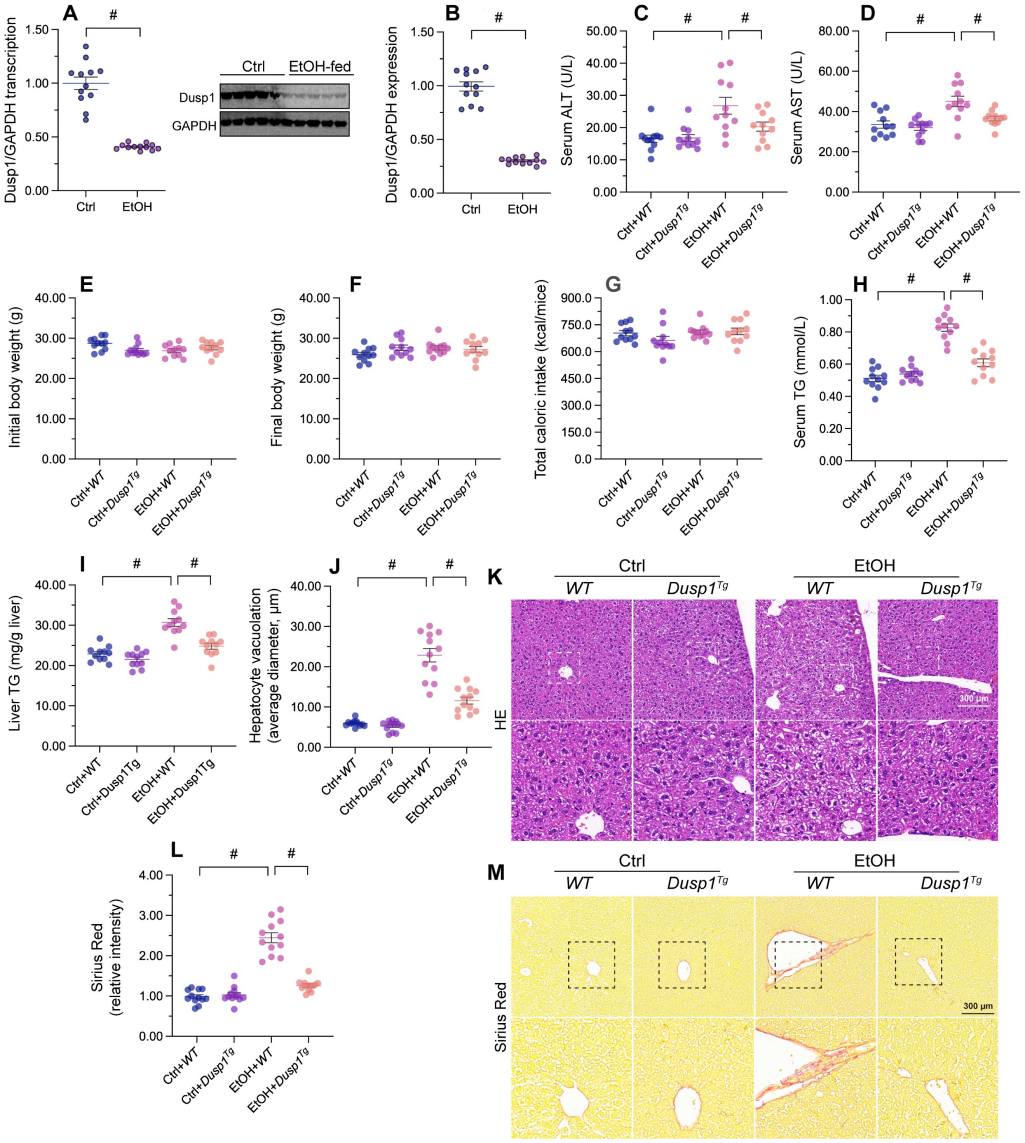
** DUSP1 overexpression attenuates alcohol-mediated hepatic dysfunction.** DUSP1 transgenic mice (*Dusp1^Tg^*) and their wild-type (WT) littermates were pair-fed control or 5% ethanol liquid diets for 8 weeks to induce alcohol-related liver disease (ALD). **(A)** Transcriptional analysis of hepatic DUSP1 expression by qPCR. **(B)** Western blot analysis of changes in DUSP1 protein levels in liver. **(C, D)** ELISA-based analysis of serum ALT and AST levels. **(E, F)** Body weight measurements in mice fed control or ethanol-containing diets. **(G)** Determination of total caloric intake. **(H, I)** ELISA-based measurements of serum and liver TG contents. **(J, K)** HE staining of liver tissues. **(L, M)** Evaluation of hepatic fibrosis via Sirius Red staining. *p<0.05.

To verify the pathological action of DUSP1 on ALD, DUSP1 mRNA and proteins levels were measured in liver tissues from ethanol-treated mice. Both transcription (Figure 1A) and protein expression (Figure 1B) of DUSP1 were significantly downregulated by alcohol treatment. To evaluate the potential impact of DUSP1 downregulation in ALD, further analyses were conducted in DUSP1 transgenic (*Dusp1^Tg^*) mice. Analysis of serum samples revealed that DUSP1 overexpression significantly reduced the secretion of alanine transaminase (ALT) and aspartate transaminase (AST) following alcohol intake (Figure 1C and 1D). However, ethanol treatment had no influence on body weight (Figure 1E and 1F) and total caloric intake (Figure 1G) in either WT or *Dusp1^Tg^* mice. Interestingly, both serum and liver triglyceride (TG) levels were markedly reduced to near-normal levels after overexpression of DUSP1 (Figure 1H and 1I). Accompanying these alterations in metabolic parameters, analysis of liver structure by HE staining showed that hepatocyte vacuolation was prominent in WT mice, whereas this phenotypic change was not evident in *Dusp1^Tg^* animals (Figure 1J and 1K). Sirius Red staining also confirmed the presence of mild hepatic fibrosis in WT but not *Dusp1^Tg^* liver tissue (Figure 1L and 1M). These data demonstrate that overexpression of DUSP1 prevents alcohol-induced hepatic dysfunction in mice.

### DUSP1 overexpression alleviates ALD-induced hepatic inflammation and oxidative stress

**Figure 2:**
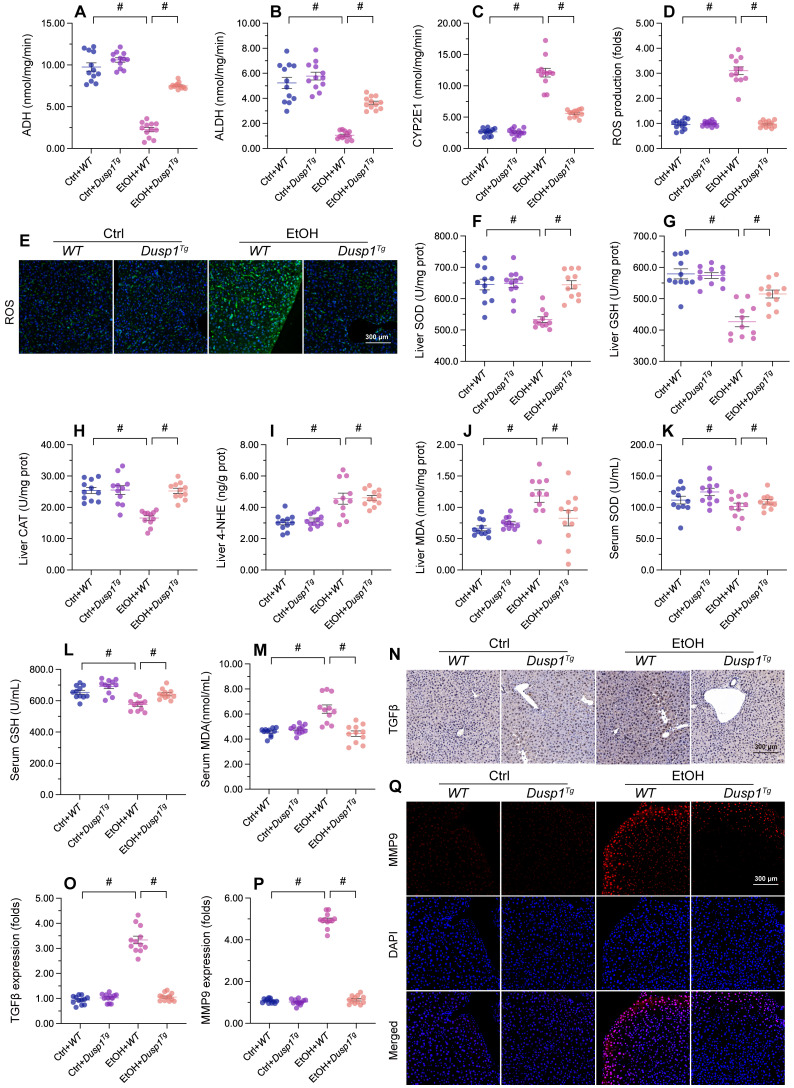
** DUSP1 overexpression alleviates ALD-induced hepatic inflammation and oxidative stress. (A-C)** ELISA-based analysis of ALDH, ADH, and CYP2E1 activity in liver tissues. **(D, E)** Evaluation of ROS production in liver sections incubated with H2DCFHDA. **(F-J)** ELISA-based analysis of SOD, GSH, CAT, MDA, and 4-NHE levels in liver tissues. **(K-M)**. ELISA-based measurements of serum SOD, GSH, and MDA levels. **(N, O)** Immunohistochemical detection of TGFβ in liver tissues. **(P, Q)** Immunofluorescence detection of MMP9 in liver tissues. *p<0.05.

Previous studies have reported that excessive alcohol intake promotes formation of acetaldehyde, which aggravates oxidative stress and inflammation in the liver 54. Two main pathways control alcohol metabolism in humans. One is the aldehyde dehydrogenase/alcohol dehydrogenase (ALDH/ADH) pathway 55 and the other is the microsomal monooxigenase system associated with cytochrome P450 2E1 (CYP2E1) 56. ELISA showed that the activity of ADH/ALDH was significantly inhibited (Figure 2A and 2B), while the content of CYP2E1 was apparently upregulated (Figure 2C), in liver tissue from alcohol-treated WT mice. However, these changes were reversed in *Dusp1^Tg^* mice.

Due to improved alcohol metabolism, the levels of ROS, generated as by-products of alcohol decomposition, were also reduced in the liver of *Dusp1^Tg^* mice (Figure 2D and 2E). As expected, the activities of antioxidant enzymes such as SOD, GSH, and CAT in liver tissue were significantly downregulated by alcohol, but attained instead near-physiological levels in the *Dusp1^Tg^* liver (Figure 2F-2H). Similarly, hepatic concentrations of lipid peroxidation products such as MDA and 4-NHE were increased by alcohol treatment in WT mice, but drastically suppressed in *Dusp1^Tg^* mice (Figure 2I and 2J). Furthermore, as a result of normalized redox biology, serum levels of SOD, GSH, and MDA were also reduced following DUSP1 overexpression (Figure 2K-2M).

Oxidative stress in hepatocytes is closely associated with hepatic inflammation. Immunohistochemistry of liver tissues showed that alcohol promoted TGFβ synthesis, and this alteration was attenuated by overexpression of DUSP1 (Figure 2N and 2O). Further evidence of the suppressive effect of DUSP1 on inflammation response in ALD was reflected by a decrease in alcohol-stimulated hepatic MMP9 expression in *Dusp1^Tg^* compared to WT mice (Figure 2P and 2Q). These data suggest that DUSP1 activity critically supports alcohol metabolism, contributing to reducing oxidative injury and preventing the inflammatory response in experimental ALD.

### DUSP1 overexpression prevents mitochondrial dysfunction in ALD

**Figure 3:**
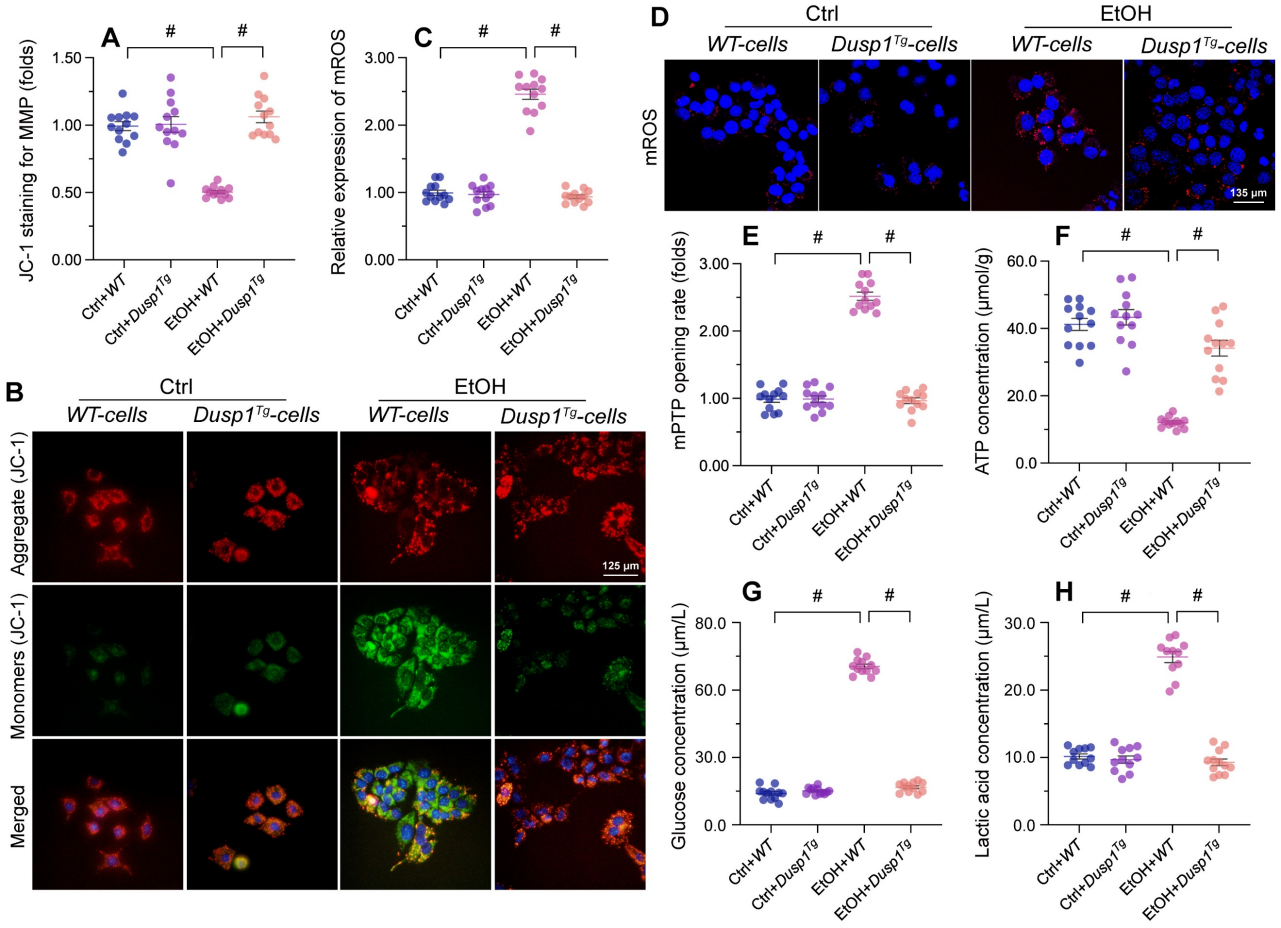
** DUSP1 overexpression prevents mitochondrial dysfunction in alcohol-treated hepatocytes.** Primary hepatocytes were isolated from WT or *Dusp1^Tg^* mice and treated with 100 mM ethanol for 24 h. **(A, B)** Analysis of mitochondrial membrane potential in primary hepatocytes loaded with JC-1. **(C, D)** Analysis of mitochondrial ROS production in primary hepatocytes loaded with mitoSOX RED. **(E)** TMRE-based analysis of mPTP opening in primary hepatocytes. **(F-H)** ELISA was used to determine the concentration of ATP, glucose, and lactic acid in hepatocyte supernatants. *p<0.05.

Mitochondria-related processes and signals are intimately involved in alcohol metabolism, ROS production, and synthesis of pro-inflammation factors. Accordingly, mitochondrial dysfunction has emerged as a major contributor to ALD pathogenesis and symptoms 11. To evaluate the potential impact of DUSP1 on mitochondrial integrity and function in the setting of ALD, we examined mitochondrial parameters in primary hepatocytes isolated from WT and *Dusp1^Tg^* mice. Following alcohol exposure, mitochondrial membrane potential (assessed by JC-1 fluorescence) was decreased in hepatocytes from WT mice, but remained unaffected in those isolated from *Dusp1^Tg^* animals (Figure 3A and 3B). Total mitochondrial ROS generation, probed with the mitochondrial superoxide sensor, was rapidly stimulated in alcohol-challenged WT hepatocytes (Figure 3C and 3D). Consistent with mitochondrial ROS overload, we detected an extended opening of the mPTP (Figure 3E). Interestingly, ROS production was relieved (Figure 3C and 3D) and the mPTP opening time was shortened (Figure 3E) in alcohol-treated *Dusp1^Tg^* hepatocytes. Further evidencing alcohol-related mitochondrial damage, in WT cells total ATP production was reduced (Figure 3F), whereas contents of glucose (Figure 3G) and lactic acid (Figure 3H) in culture media were increased. In contrast, following alcohol treatment, ATP synthesis and glucose/lactic acid levels were largely normalized in DUSP1-overexpressing hepatocytes.

### DUSP1 overexpression restores mitophagy in alcohol-treated hepatocytes

**Figure 4:**
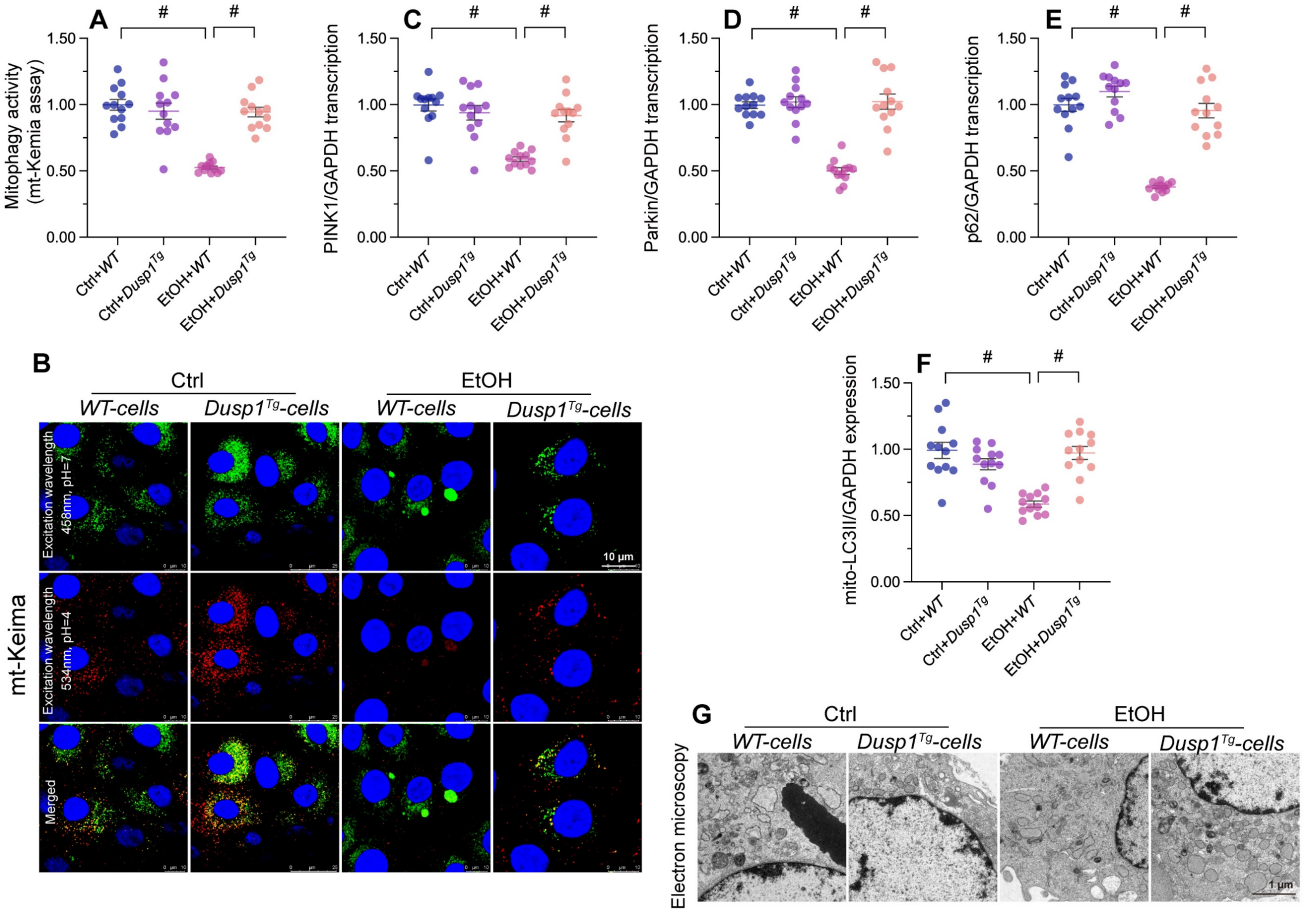
**DUSP1 overexpression restores mitophagy in alcohol-treated hepatocytes. (A, B)** Evaluation of mitophagic activity in primary hepatocytes (mito-Keima assay). **(C-E)** Transcriptional analysis of PINK1, Parkin, and p62 expression in primary hepatocytes using qPCR. **(F)** Western blot analysis of mito-LC3II expression in primary hepatocytes. **(G)** Electron microscopy was applied to evaluate the formation of autophagolysosomes. *p<0.05.

Mitophagy constitutes a protective mechanism against alcohol-mediated mitochondrial damage. *In vitro* assays in primary hepatocytes transfected with the mitophagy indicator mito-Keima showed that mitophagic flux was significantly inhibited by alcohol treatment in WT cells, but restored to near-normal levels after overexpression of DUSP1 (Figure 4A and 4B). In agreement with this finding, the transcription of PINK1 and Parkin, two mitophagy-associated proteins, and that of p62, a major autophagosome cargo protein, was downregulated in alcohol-exposed WT hepatocytes and reversed to near-normal levels in *Dusp1^Tg^* hepatocytes (Figure 4C-4E). Subsequently, mitochondria were isolated from hepatocytes and the expression of mitochondria-localized LC3II (mito-LC3II), an autophagosome marker, was analyzed through western blots. The results illuminated that alcohol drastically suppressed the levels of mito-LC3II, while this effect was reversed by DUSP1 overexpression (Figure 4F). Lastly, electron microscopy was used to explore the formation of autophagolysosomes. As shown in Figure 4G, negligible evidence of autophagolysosome formation was obtained in alcohol-treated WT hepatocytes. By comparison, autophagolysosome formation was clearly evident in *Dusp1^Tg^* hepatocytes. These data illustrated that DUSP1 overexpression reverses alcohol-related mitophagy inhibition in hepatocytes.

### Mitophagy inhibition abolishes the *in vivo* and *in vitro* protective effects of DUSP1 overexpression

**Figure 5:**
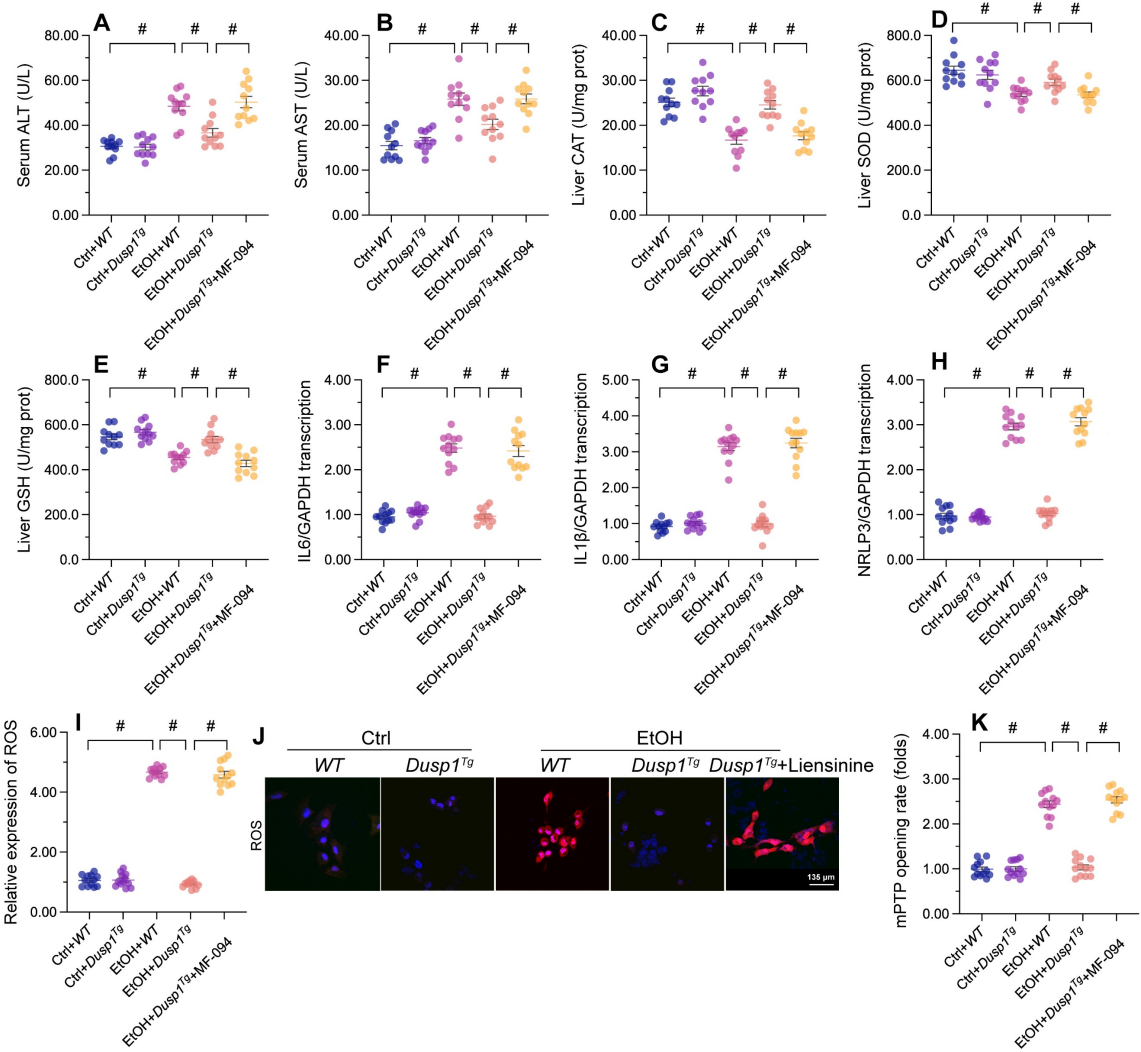
** Mitophagy inhibition abolishes DUSP1-mediated hepatocyte protection *in vivo* and *in vitro*.** To inhibit mitophagic activity, mice or primary hepatocytes were treated with liensinine. **(A, B)** ELISA-based analysis of serum ALT and AST contents. **(C-E)** ELISA-based determinations of SOD, GSH, and CAT levels in liver tissues. **(F-H)** Transcriptional analysis of hepatic IL6, NLRP3, and IL1β expression by qPCR. **(I, J)** Analysis of alterations in mitochondrial membrane potential in JC-1-loaded primary hepatocytes. **(K, L)** Evaluation of cellular ROS production in primary hepatocytes loaded with DHE. **(M)** TMRE-based analysis of mPTP opening in primary hepatocytes. *p<0.05.

To assess whether mitophagy is implicated in the protective effects of DUSP1 against ALD, liensinine, a mitophagy antagonist, was administered to WT and *Dusp1^Tg^* mice before alcohol treatment. As shown in Figure 5A and 5B, following alcohol treatment and compared to WT mice, *Dusp1^Tg^* mice showed decreased serum levels of ALT and AST, and this effect was negated by liensinine4 administration. In addition, the anti-oxidative effect of DUSP1 overexpression in alcohol-treated mice, denoted by restored expression of hepatic SOD, GSH, and CAT enzymes, was abolished upon liensinine administration (Figure 5C-5E). Furthermore, liensinine treatment reversed DUSP1 overexpression-induced repression of alcohol-induced pro-inflammatory gene transcription in the livers of *Dusp1^Tg^* mice (Figure 5F-5H).

Further suggesting a critical hepatoprotective role of DUSP1-stimulated mitophagy activation in ALD, the stabilizing effect of DUSP1 overexpression on intracellular ROS production (Figure 5I and 5J) and mPTP opening time (Figure 5K) was attenuated or nullified after liensinine administration in cultured primary hepatocytes. These results are robust evidence that mitophagy induction underlies DUSP1-afforded hepatoprotection against ALD.

### DUSP1 interacts with and prevents CUL1 nuclear accumulation

**Figure 6:**
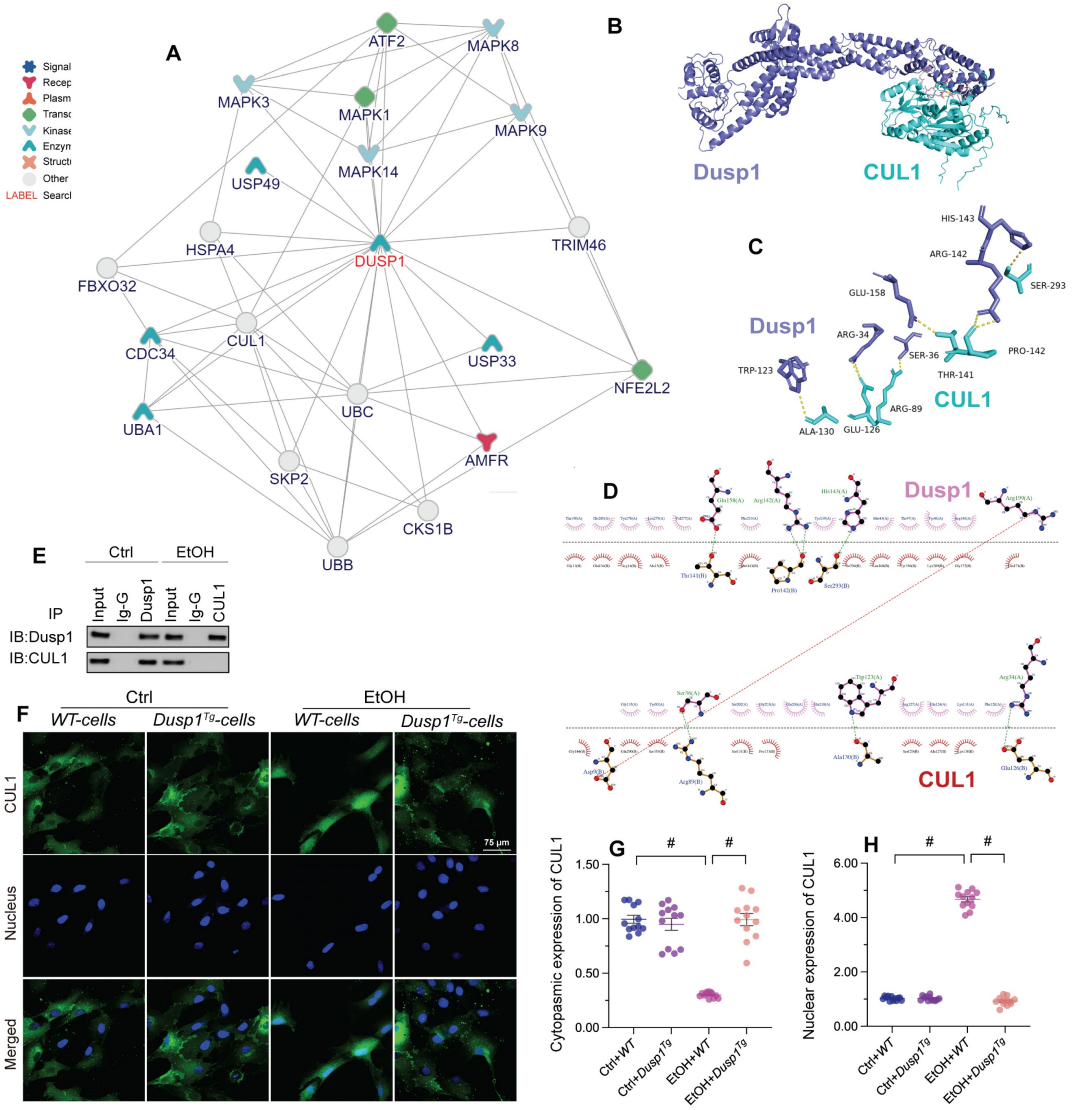
**DUSP1 interacts with and prevents CUL1 nuclear import. (A)** Predicted interactions between DUSP1 and other proteins, based on analysis of the inBio Discover database. **(B-D)** Molecular docking analysis of DUSP1 and CUL1. **(E)** Co-IP assay results indicating the interaction between DUSP1 and CUL1. **(F-H)** Immunofluorescence detection of CUL1 expression in hepatocytes in response to alcohol treatment or DUSP1 overexpression. *p<0.05.

To evaluate the molecular mechanisms underlying DUSP1-related hepatoprotection in ALD, we explored predicted protein-protein interactions for DUSP1 using the inBio Discover database (https://inbio-discover.com). The protein-protein interaction network related to DUSP1 is shown in Figure 6A. Among these proteins, CUL1 has been identified as a key player in Parkin-related mitophagy 31, 32. Therefore, we asked whether DUSP1 modulates mitophagy through CUL1. To address this question, we first asserted the DUSP1/CUL1 interaction through molecular docking analysis, which revealed the interacting sites illustrated in Figure 6B. Further, through amino acid sequence analysis we defined the active region that is required for DUSP1/CUL1 association (Figure 6C and 6D). Subsequently, Co-IP assays confirmed the endogenous interaction between these proteins in hepatocytes under control conditions (Figure 6E). However, consistent with the drop induced by alcohol on DUSP1 protein expression, the binding of DUSP1 to CUL1 was markedly inhibited after alcohol treatment (Figure 6E).

Recent studies indicated that CUL1 contributes to inhibition of the transcription of mitochondria-related genes 57, 58. Therefore, we asked whether alcohol-mediated DUSP1 downregulation and the ensuing DUSP1/CUL1 disassociation contribute to CUL1 nuclear translocation and CUL1-mediated interruption of mitophagy-related gene transcription. Immunofluorescence showed that under control conditions CUL1 is mainly located in the cytoplasm (Figure 6F-6H). However, after exposure to alcohol the expression of cytoplasmic CUL1 was significantly reduced, along with a rise in its nuclear expression. In turn, in the presence of alcohol DUSP1 overexpression sustained CUL1 cytoplasmic expression and inhibited its nuclear translocation (Figure 6F-6H). In sum, these findings indicate that alcohol-mediated DUSP1 downregulation leads to DUSP1/CUL1 dissociation and promotes the translocation of CUL1 into the cell nucleus.

### Loss of CUL1 promotes p62 transcription and activates p62-related mitophagy

**Figure 7:**
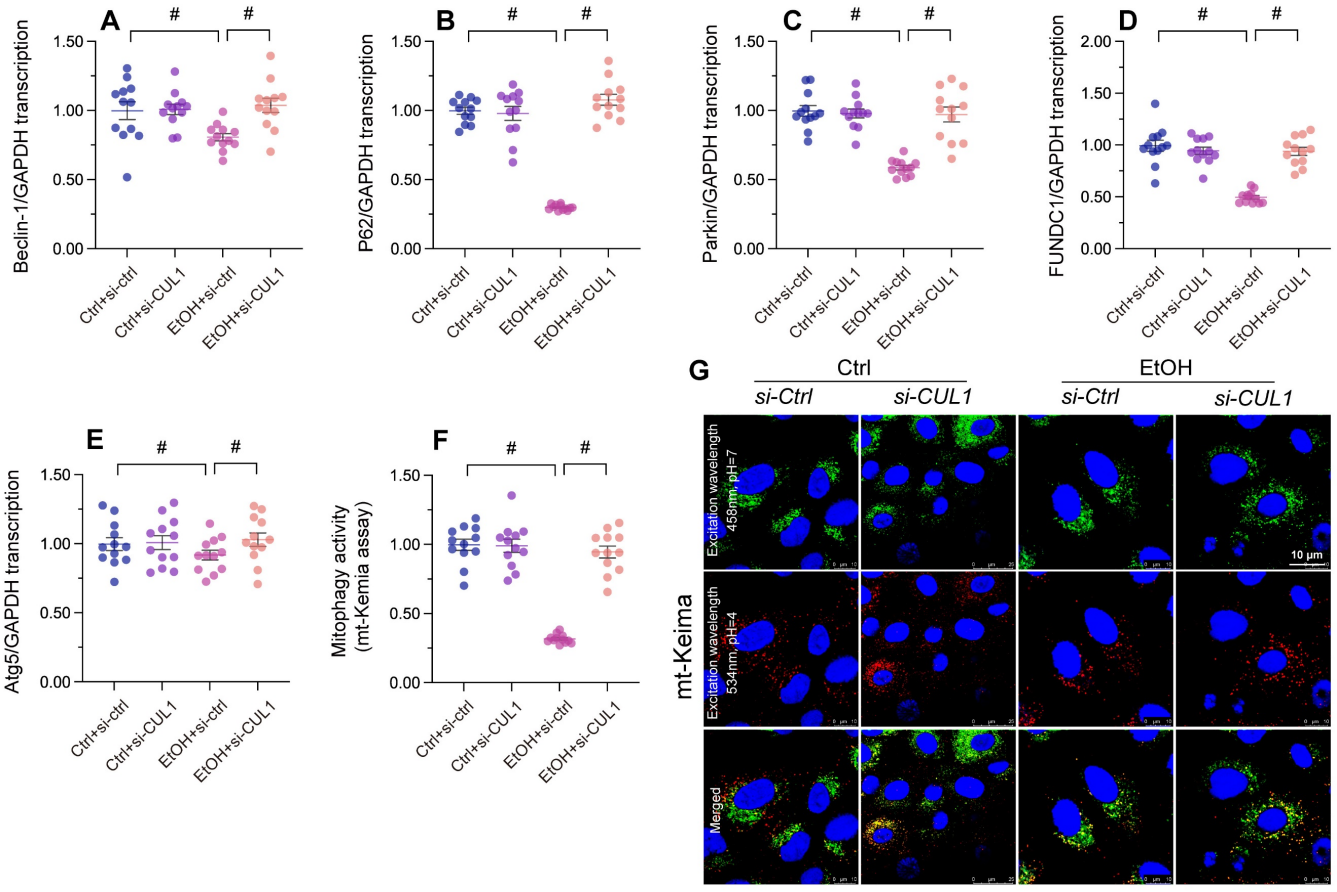
**Loss of CUL1 augments p62 transcription and activates p62-related mitophagy.** Negative control siRNA or siRNA targeting CUL1 (siRNA-CUL1) was transfected into primary hepatocytes before alcohol treatment. **(A-E)** Transcriptional analysis of Atg5, p62, Beclin-1, Fundc1, and Parkin expression by qPCR. **(F, G)** Evaluation of mitophagic activity in primary hepatocytes (mito-Keima assay). *p<0.05.

To analyze the effect of alcohol-induced CUL1 nuclear accumulation on mitophagic activity, siRNA against CUL1 was transfected into primary hepatocytes before alcohol treatment. As shown in Figure 7A-7E, alcohol exposure significantly reduced the transcription of Atg5, p62, Beclin-1, Fundc1, and Parkin in hepatocytes transfected with negative control siRNA. In contrast, upon transfection with CUL1-siRNA, the transcription of p62 and Parkin in alcohol-treated cells was significantly upregulated, whereas that of Atg5, Fundc1, and Beclin-1 was not affected. These data suggest that CUL-1 suppresses mitophagy through transcriptional repression of p62 and Parkin. Furthermore, in CUL-1 knockdown cells the transcriptional upregulation of p62 was more prominent than that of Parkin, suggesting that CUL-1-dependent mitophagy inhibition is mainly determined by blunted p62 expression. Accordingly, mito-Keima assays showed that following alcohol exposure mitophagic activity was restored to control levels in CUL1-siRNA-transfected cells (Figure 7F and 7G). Thus, these data indicate that CUL-1 inhibition sustains mitophagy in alcohol-treated hepatocytes.

### CUL1 deficiency maintains mitochondrial integrity and hepatocyte function upon alcohol treatment

**Figure 8:**
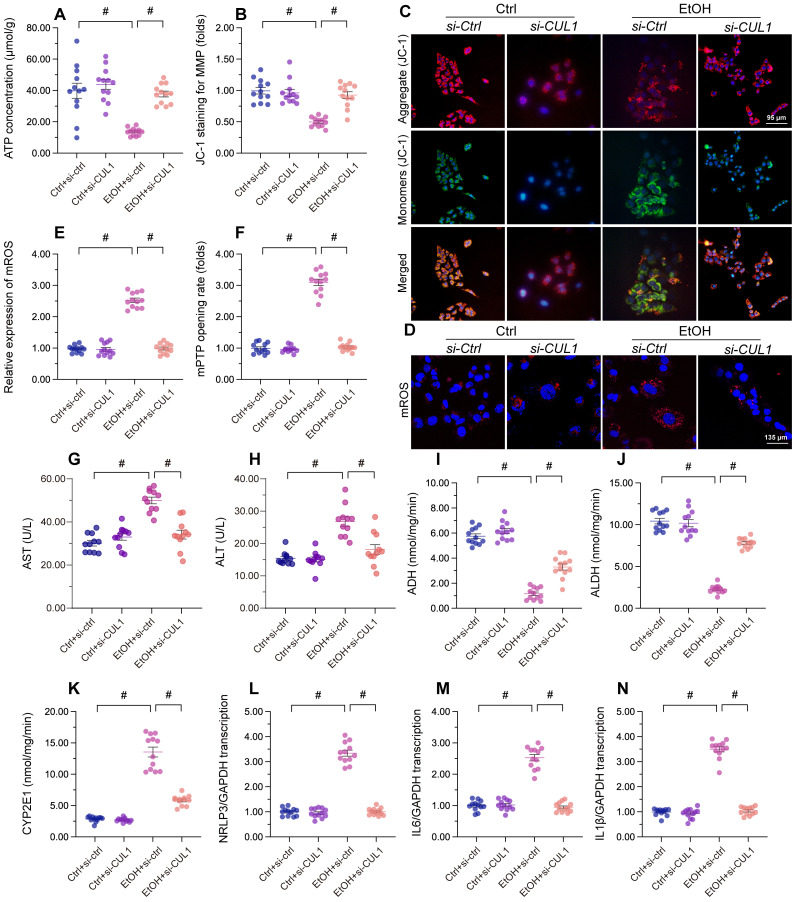
**CUL1 deficiency sustains mitochondrial integrity and hepatocyte function upon alcohol treatment.** Negative control siRNA and siRNA targeting CUL1 (siRNA-CUL1) were transfected into primary hepatocytes before alcohol treatment. **(A)** ELISA-based analysis of ATP production in primary hepatocytes. **(B, C)** Analysis of alterations in mitochondrial membrane potential in primary hepatocytes loaded with JC-1. **(D, E)** Evaluation of mitochondrial ROS production in primary hepatocytes loaded with mitoSOX RED. **(F)** TMRE-based analysis of mPTP opening in primary hepatocytes **(G, H)** ELISA-based determinations of ALT and AST levels in the media of cultured hepatocytes **(I-K)** ELISA-based analysis of the activities of ALDH, ADH, and CYP2E1 in primary hepatocytes. **(L-N)** Transcriptional analysis of IL6, NLRP3, and IL1β expression in primary hepatocytes by qPCR. *p<0.05.

Lastly, we asked whether CUL1deficiency has protective effects in alcohol-treated hepatocytes. Our assays showed that alcohol-suppressed ATP production was restored by CUL1-siRNA transfection (Figure 8A), indicating that CUL1 deletion favored mitochondrial metabolism. Consistent with this finding, CUL1 knockdown also reduced or prevented alcohol-induced mitochondrial membrane potential depolarization (Figure 8B and 8C), mitochondrial ROS production (Figure 8D and 8E), and mPTP opening (Figure 8F). Owing to its protective influence on mitochondrial function, CUL1 knockdown was associated also with decreased ALT and AST release from hepatocytes (Figure 8G and 8H). Furthermore, CUL1 silencing restored the activities of ALDH/ADH and reduced the content of CYP2E1 in alcohol-treated hepatocytes (Figure 8I-8K). Lastly, we found that CUL1 knockdown also repressed alcohol-induced pro-inflammatory gene transcription (Figure 8L-8N). The above findings demonstrate that CUL1 deficiency preserves mitochondrial function and protects hepatic cells against alcoholic injury.

## Discussion

Following on our previous reports 39, the present study explored the molecular mechanisms underlying alcohol-related liver injury. We identified DUSP1 downregulation as a likely initial signal in ALD development, contributing to mitochondrial dysfunction via mitophagy inhibition. We reveal that under physiological conditions, DUSP1 binds to CUL1 in the cytosol and prevents its translocation into the nucleus. After exposure to alcohol, the expression of DUSP1 is reduced, leading to CUL1 disassociation and shuttling into the nucleus, where it acts as a transcriptional inhibitor to suppress the expression of Parkin and p62. The latter is associated with a drop in mitophagic activity, resulting in impaired recycling of the dysfunctional mitochondrial fraction and impaired ATP generation. Accordingly, mitophagy restoration via DUSP1 overexpression or CUL1 depletion greatly attenuated mitochondrial dysfunction in alcohol-challenged hepatocytes, as evidenced by normalized ATP production, restored mitochondrial membrane potential, and inhibition of cellular oxidative stress. Consistent with the fundamental role played by mitochondria in alcohol metabolism, reduced levels of alcohol-metabolizing enzymes, along with enhanced oxidative stress and pro-inflammatory gene expression, were observed in alcohol-challenged hepatocytes. We thus conclude that alcohol-mediated DUSP1 downregulation highly promoted the progression of ALD by inhibiting mitophagy and hence promoting mitochondrial dysfunction. Based on these data, the DUSP1/CUL1/p62 pathway arises as a major mechanism influencing alcohol-related hepatic damage. Accordingly, inhibition of DUSP1 downregulation and/or CUL1 nuclear translocation may represent promising therapeutic approaches to restore mitochondrial fitness and hepatocyte homeostasis in ALD patients.

Several studies highlighted a close relationship between DUSP1 and liver diseases. In its role as a tumor suppressor, DUSP1-mediated inhibition of p53 activity has been reported to delay the progression of hepatocellular carcinoma 59. Meanwhile, mild downregulation of DUSP1 protein expression was reported in patients with non-alcoholic fatty liver disease (NAFLD) who underwent laparoscopic sleeve gastrectomy 60. Furthermore, DUSP1 downregulation was associated with activation of MAPK/p38 and MAPK/JNK signaling and accelerated development of hepatosteatosis in NAFLD patients 60. Transcriptional profiling using cDNA microarrays showed that DUSP1 mRNA expression in liver is strongly upregulated and attenuates pro-apoptotic signal induction thorough the JNK pathway during the ischemia/reperfusion period following liver transplantation 61. In line with these observations, our animal experiments and cellular studies identified DUSP1 downregulation in hepatocytes as an important event in response to alcohol treatment. Although we have not established the dose- or time-response relationship between alcohol intake/exposure and DUSP1 downregulation levels, it is reasonable to predict that lower DUSP1 expression will be associated with worse liver function.

The hepatoprotective action offered by DUSP1 in ALD is attributable to mitochondrial protection. Mouse experiments have illustrated that DUSP1 interferes with mitochondrial fission and thus blocks mitochondria-dependent cardiomyocyte death during myocardial ischemia-reperfusion injury 29. Another study reported the anti-oxidative effects of DUSP1, related to improved mitochondrial metabolism and reduced mitochondrial superoxide leakage, in the mouse cochlea 62. Recent studies revealed that DUSP1 positively modulates Parkin-related mitophagy during diabetic nephropathy 30, although the underlying molecular basis has not been clearly demonstrated. The results of the present study provide a plausible explanation for the above observations, by showing that DUSP1 overexpression rescues mitophagic activity in alcohol-challenged hepatocytes through preventing CUL1-related p62 transcriptional repression. Based on these results, DUSP1 emerges as a positive regulator of mitophagy by retaining cytosolic CUL1 to sustain p62 transcription. This finding thus provides a new piece of evidence on the multiple beneficial effects of DUSP1 in preserving mitochondrial integrity.

Compared to DUSP1, research on CUL1 is scarcer. Hence, except for liver cancer, its potential involvement in liver diseases remains fairly unexplored. The expression of CUL1 is markedly upregulated in patients with hepatocellular carcinoma, and higher CUL1 levels are closely linked to poor 5-year overall survival rates 63. Meanwhile, hinting at the important influence of CUL1 on cell metabolism, pharmacological inhibition of CUL1 neddylation has been reported to reduce hyperglycemia through sensitizing hepatic insulin signaling in mice 64. In our study, we found that CUL1 expression was significantly elevated in liver tissue from alcohol-treated mice. Importantly, in agreement with recent evidence indicating that nuclear CUL1 accumulation represses the expression of genes related to mitochondrial metabolism 36, 44, 45, 65, 66, our study reveals that upon nuclear translocation, CUL1 functions as a transcriptional inhibitor of p62 and Parkin during ALD development. However, additional experiments are still needed to explore whether CUL1 directly binds to the transcriptional promoters of p62 and Parkin, or indirectly controls p62/Parkin mRNA expression with the assistance of transcriptional factors.

## Conclusions

In sum, our experiments shed light on pathogenesis of ALD by demonstrating that alcohol-mediated DUSP1 downregulation contributes to its disassociation from CUL1 in the cytoplasm. This results in the shuttling of CUL1 into the nucleus, where CUL1 serves as a transcriptional repressor of p62/Parkin expression, leading to blunted mitophagy activation. As a result, damaged mitochondria accumulate in hepatocytes, determining impaired alcohol metabolism, enhanced ROS production, increased inflammatory response, hepatocyte vacuolation, and hepatic fibrosis and dysfunction. Therefore, our study identified the DUSP1/CUL1/p62 axis as a novel stress signaling mechanism differentially modulated in the liver in response to alcohol exposure. This evidence may lay the groundwork for a more complete understanding of the pathogenesis of ALD and the development of targeted drugs for clinical management.

## References

[1] Tan Y, Xi D, Cai C, Jiang X, Chen S, Hu R (2022). DUSP1 overexpression attenuates septic cardiomyopathy through reducing VCP phosphorylation and normalizing mitochondrial quality control. Acta Pharmaceutica Sinica B.

[2] Li Y, Xu S, Xu Q, Chen Y (2020). Clostridium difficile toxin B induces colonic inflammation through the TRIM46/DUSP1/MAPKs and NF-κB signalling pathway. Artif Cells Nanomed Biotechnol.

[3] Moosavi SM, Prabhala P, Ammit AJ (2017). Role and regulation of MKP-1 in airway inflammation. Respir Res.

[4] Hammer M, Echtenachter B, Weighardt H, Jozefowski K, Rose-John S, Männel DN (2010). Increased inflammation and lethality of Dusp1-/- mice in polymicrobial peritonitis models. Immunology.

[5] Xin Y, Tang L, Chen J, Chen D, Wen W, Han F (2021). Inhibition of miR-101-3p protects against sepsis-induced myocardial injury by inhibiting MAPK and NF-κB pathway activation via the upregulation of DUSP1. Int J Mol Med.

[6] Liu LP, Zhang J, Pu B, Li WQ, Wang YS (2020). Upregulation of JHDM1D-AS1 alleviates neuroinflammation and neuronal injury via targeting miR-101-3p-DUSP1 in spinal cord after brachial plexus injury. Int Immunopharmacol.

[7] Qu H, Liu S, Cheng C, Zhao H, Gao X, Wang Z (2020). Hepatoprotection of pine nut polysaccharide via NRF2/ARE/MKP1/JNK signaling pathways against carbon tetrachloride-induced liver injury in mice. Food Chem Toxicol.

[8] Sha J, Zhang H, Zhao Y, Feng X, Hu X, Wang C (2019). Dexmedetomidine attenuates lipopolysaccharide-induced liver oxidative stress and cell apoptosis in rats by increasing GSK-3β/MKP-1/Nrf2 pathway activity via the α2 adrenergic receptor. Toxicol Appl Pharmacol.

[9] Sha J, Feng X, Chen Y, Zhang H, Li B, Hu X (2019). Dexmedetomidine improves acute stress-induced liver injury in rats by regulating MKP-1, inhibiting NF-κB pathway and cell apoptosis. J Cell Physiol.

[10] Luo L, Chen Y, Wang H, Wang S, Liu K, Li X (2018). Mkp-1 protects mice against toxin-induced liver damage by promoting the Nrf2 cytoprotective response. Free Radic Biol Med.

[11] Hoek JB, Cahill A, Pastorino JG (2002). Alcohol and mitochondria: a dysfunctional relationship. Gastroenterology.

[12] Nassir F, Ibdah JA (2014). Role of mitochondria in alcoholic liver disease. World J Gastroenterol.

[13] Hao L, Sun Q, Zhong W, Zhang W, Sun X, Zhou Z (2018). Mitochondria-targeted ubiquinone (MitoQ) enhances acetaldehyde clearance by reversing alcohol-induced posttranslational modification of aldehyde dehydrogenase 2: A molecular mechanism of protection against alcoholic liver disease. Redox Biol.

[14] Silva J, Spatz MH, Folk C, Chang A, Cadenas E, Liang J (2021). Dihydromyricetin improves mitochondrial outcomes in the liver of alcohol-fed mice via the AMPK/Sirt-1/PGC-1α signaling axis. Alcohol.

[15] Zhao H, Liu S, Zhao H, Liu Y, Xue M, Zhang H (2021). Protective effects of fucoidan against ethanol-induced liver injury through maintaining mitochondrial function and mitophagy balance in rats. Food Funct.

[16] Zhou H, Dai Z, Li J, Wang J, Zhu H, Chang X (2023). TMBIM6 prevents VDAC1 multimerization and improves mitochondrial quality control to reduce sepsis-related myocardial injury. Metabolism.

[17] Zou R, Tao J, Qiu J, Lu H, Wu J, Zhu H (2022). DNA-PKcs promotes sepsis-induced multiple organ failure by triggering mitochondrial dysfunction. Journal of Advanced Research.

[18] Zou R, Shi W, Qiu J, Zhou N, Du N, Zhou H (2022). Empagliflozin attenuates cardiac microvascular ischemia/reperfusion injury through improving mitochondrial homeostasis. Cardiovasc Diabetol.

[19] Wang S, Zhu H, Li R, Mui D, Toan S, Chang X (2022). DNA-PKcs interacts with and phosphorylates Fis1 to induce mitochondrial fragmentation in tubular cells during acute kidney injury. Sci Signal.

[20] Sun D, Wang J, Toan S, Muid D, Li R, Chang X (2022). Molecular mechanisms of coronary microvascular endothelial dysfunction in diabetes mellitus: focus on mitochondrial quality surveillance. Angiogenesis.

[21] Durcan TM, Fon EA (2015). The three 'P's of mitophagy: PARKIN, PINK1, and post-translational modifications. Genes Dev.

[22] Ma L, Zou R, Shi W, Zhou N, Chen S, Zhou H (2022). SGLT2 inhibitor dapagliflozin reduces endothelial dysfunction and microvascular damage during cardiac ischemia/reperfusion injury through normalizing the XO-SERCA2-CaMKII-coffilin pathways. Theranostics.

[23] Chang X, Li Y, Cai C, Wu F, He J, Zhang Y (2022). Mitochondrial quality control mechanisms as molecular targets in diabetic heart. Metabolism.

[24] Zhu H, Toan S, Mui D, Zhou H (2021). Mitochondrial quality surveillance as a therapeutic target in myocardial infarction. Acta Physiol (Oxf).

[25] Zhou H, Zhu P, Wang J, Toan S, Ren J (2019). DNA-PKcs promotes alcohol-related liver disease by activating Drp1-related mitochondrial fission and repressing FUNDC1-required mitophagy. Signal Transduct Target Ther.

[26] Zhou T, Chang L, Luo Y, Zhou Y, Zhang J (2019). Mst1 inhibition attenuates non-alcoholic fatty liver disease via reversing Parkin-related mitophagy. Redox Biol.

[27] Lu X, Xuan W, Li J, Yao H, Huang C, Li J (2021). AMPK protects against alcohol-induced liver injury through UQCRC2 to up-regulate mitophagy. Autophagy.

[28] Chao X, Ding WX (2019). Role and mechanisms of autophagy in alcohol-induced liver injury. Adv Pharmacol.

[29] Jin Q, Li R, Hu N, Xin T, Zhu P, Hu S (2018). DUSP1 alleviates cardiac ischemia/reperfusion injury by suppressing the Mff-required mitochondrial fission and Bnip3-related mitophagy via the JNK pathways. Redox Biol.

[30] Lu C, Wu B, Liao Z, Xue M, Zou Z, Feng J (2021). DUSP1 overexpression attenuates renal tubular mitochondrial dysfunction by restoring Parkin-mediated mitophagy in diabetic nephropathy. Biochem Biophys Res Commun.

[31] Staropoli JF, McDermott C, Martinat C, Schulman B, Demireva E, Abeliovich A (2003). Parkin is a component of an SCF-like ubiquitin ligase complex and protects postmitotic neurons from kainate excitotoxicity. Neuron.

[32] Choo YS, Vogler G, Wang D, Kalvakuri S, Iliuk A, Tao WA (2012). Regulation of parkin and PINK1 by neddylation. Hum Mol Genet.

[33] Michail O, Moris D, Theocharis S, Griniatsos J (2018). Cullin-1 and -2 Protein Expression in Colorectal Cancer: Correlation with Clinicopathological Variables. *In vivo*.

[34] Zhou YH, Xia J, Xu WH, Zhu X, Wu XH, Hua D (2016). Cullin-1 promotes cell proliferation in human breast cancer and is related to diabetes. Int J Biol Markers.

[35] Jiang H, He D, Xu H, Liu J, Qu L, Tong S (2015). Cullin-1 promotes cell proliferation via cell cycle regulation and is a novel in prostate cancer. Int J Clin Exp Pathol.

[36] Sweeney MA, Iakova P, Maneix L, Shih FY, Cho HE, Sahin E (2020). The ubiquitin ligase Cullin-1 associates with chromatin and regulates transcription of specific c-MYC target genes. Sci Rep.

[37] Bramasole L, Sinha A, Gurevich S, Radzinski M, Klein Y, Panat N (2019). Proteasome lid bridges mitochondrial stress with Cdc53/Cullin1 NEDDylation status. Redox Biol.

[38] Noda S, Sato S, Fukuda T, Ueno S, Tada N, Hattori N (2022). Impaired mitochondrial accumulation and Lewy pathology in neuron-specific FBXO7-deficient mice. Mol Brain.

[39] Zhou H, Zhu P, Wang J, Toan S, Ren J (2019). DNA-PKcs promotes alcohol-related liver disease by activating Drp1-related mitochondrial fission and repressing FUNDC1-required mitophagy. Signal transduction and targeted therapy.

[40] Xu H, Wan X-d, Zhu R-r, Liu J-l, Liu J-c, Zhou X-l (2022). Keap-NRF2 signaling contributes to the Notch1 protected heart against ischemic reperfusion injury via regulating mitochondrial ROS generation and bioenergetics. International Journal of Biological Sciences.

[41] Xiang Z, Huang G, Wu H, He Q, Yang C, Dou R (2022). SNHG16 upregulation-induced positive feedback loop with YAP1/TEAD1 complex in Colorectal Cancer cell lines facilitates liver metastasis of colorectal cancer by modulating CTCs epithelial-mesenchymal transition. International Journal of Biological Sciences.

[42] Zhou H, Toan S, Zhu P, Wang J, Ren J, Zhang Y (2020). DNA-PKcs promotes cardiac ischemia reperfusion injury through mitigating BI-1-governed mitochondrial homeostasis. Basic Res Cardiol.

[43] Wang J, Zhu P, Li R, Ren J, Zhang Y, Zhou H (2020). Bax inhibitor 1 preserves mitochondrial homeostasis in acute kidney injury through promoting mitochondrial retention of PHB2. Theranostics.

[44] Wang Y, Jasper H, Toan S, Muid D, Chang X, Zhou H (2021). Mitophagy coordinates the mitochondrial unfolded protein response to attenuate inflammation-mediated myocardial injury. Redox Biol.

[45] Zhu H, Tan Y, Du W, Li Y, Toan S, Mui D (2021). Phosphoglycerate mutase 5 exacerbates cardiac ischemia-reperfusion injury through disrupting mitochondrial quality control. Redox Biol.

[46] Wang J, Zhu P, Li R, Ren J, Zhou H (2020). Fundc1-dependent mitophagy is obligatory to ischemic preconditioning-conferred renoprotection in ischemic AKI via suppression of Drp1-mediated mitochondrial fission. Redox Biol.

[47] Jiang J, Ding Y, Lu J, Chen Y, Chen Y, Zhao W (2022). Integrative analysis reveals a clinicogenomic landscape associated with liver metastasis and poor prognosis in hepatoid adenocarcinoma of the stomach. International Journal of Biological Sciences.

[48] Guo Z, Tuo H, Tang N, Liu FY, Ma SQ, An P (2022). Neuraminidase 1 deficiency attenuates cardiac dysfunction, oxidative stress, fibrosis, inflammatory via AMPK-SIRT3 pathway in diabetic cardiomyopathy mice. Int J Biol Sci.

[49] Huang G, Xiang Z, Wu H, He Q, Dou R, Lin Z (2022). The lncRNA BDNF-AS/WDR5/FBXW7 axis mediates ferroptosis in gastric cancer peritoneal metastasis by regulating VDAC3 ubiquitination. Int J Biol Sci.

[50] Tan Y, Mui D, Toan S, Zhu P, Li R, Zhou H (2020). SERCA Overexpression Improves Mitochondrial Quality Control and Attenuates Cardiac Microvascular Ischemia-Reperfusion Injury. Mol Ther Nucleic Acids.

[51] Wang J, Toan S, Li R, Zhou H (2020). Melatonin fine-tunes intracellular calcium signals and eliminates myocardial damage through the IP3R/MCU pathways in cardiorenal syndrome type 3. Biochem Pharmacol.

[52] Dong Y, Fan H, Zhang Z, Jiang F, Li M, Zhou H (2022). Berberine ameliorates DSS-induced intestinal mucosal barrier dysfunction through microbiota-dependence and Wnt/β-catenin pathway. Int J Biol Sci.

[53] Zhang H, Liu J, Zhou Y, Qu M, Wang Y, Guo K (2022). Neutrophil extracellular traps mediate m(6)A modification and regulates sepsis-associated acute lung injury by activating ferroptosis in alveolar epithelial cells. Int J Biol Sci.

[54] Campollo O (2019). Alcohol and the Liver: The Return of the Prodigal Son. Ann Hepatol.

[55] Ehlers CL, Liang T, Gizer IR (2012). ADH and ALDH polymorphisms and alcohol dependence in Mexican and Native Americans. Am J Drug Alcohol Abuse.

[56] Lu Y, Cederbaum AI (2008). CYP2E1 and oxidative liver injury by alcohol. Free radical biology & medicine.

[57] Sweeney MA, Iakova P, Maneix L, Shih F-Y, Cho HE, Sahin E (2020). The ubiquitin ligase Cullin-1 associates with chromatin and regulates transcription of specific c-MYC target genes. Scientific Reports.

[58] Zhou Q, Zheng Y, Sun Y (2021). Neddylation regulation of mitochondrial structure and functions. Cell & Bioscience.

[59] Hao PP, Li H, Lee MJ, Wang YP, Kim JH, Yu GR (2015). Disruption of a regulatory loop between DUSP1 and p53 contributes to hepatocellular carcinoma development and progression. J Hepatol.

[60] Zhang TT, Wang Y, Zhang XW, Yang KY, Miao XQ, Zhao GH (2022). MiR-200c-3p Regulates DUSP1/MAPK Pathway in the Nonalcoholic Fatty Liver After Laparoscopic Sleeve Gastrectomy. Front Endocrinol (Lausanne).

[61] Boutros T, Nantel A, Emadali A, Tzimas G, Conzen S, Chevet E (2008). The MAP kinase phosphatase-1 MKP-1/DUSP1 is a regulator of human liver response to transplantation. Am J Transplant.

[62] Bermúdez-Muñoz JM, Celaya AM, García-Mato Á, Muñoz-Espín D, Rodríguez-de la Rosa L, Serrano M (2021). Dual-Specificity Phosphatase 1 (DUSP1) Has a Central Role in Redox Homeostasis and Inflammation in the Mouse Cochlea. Antioxidants (Basel).

[63] Liu W, Wang Y, Zhang C, Huang B, Bai J, Tian L (2015). Cullin1 is up-regulated and associated with poor patients' survival in hepatocellular carcinoma. Int J Clin Exp Pathol.

[64] Chen C, Gu L, Matye DJ, Clayton YD, Hasan MN, Wang Y (2022). Cullin neddylation inhibitor attenuates hyperglycemia by enhancing hepatic insulin signaling through insulin receptor substrate stabilization. Proc Natl Acad Sci U S A.

[65] Zhou H, Ren J, Toan S, Mui D (2021). Role of mitochondrial quality surveillance in myocardial infarction: From bench to bedside. Ageing Res Rev.

[66] Chang X, Lochner A, Wang HH, Wang S, Zhu H, Ren J (2021). Coronary microvascular injury in myocardial infarction: perception and knowledge for mitochondrial quality control. Theranostics.

